# A general method for the synthesis of covalent and ionic amine borane complexes containing trinitromethyl fragments†

**DOI:** 10.1039/d1ra00440a

**Published:** 2021-03-05

**Authors:** Jin Wang, Ming-Yue Ju, Xi-Meng Chen, Xuenian Chen

**Affiliations:** School of Chemistry and Chemical Engineering, Henan Key Laboratory of Boron Chemistry and Advanced Energy Materials, Henan Normal University Xinxiang Henan 453007 China xnchen@htu.edu.cn chenximeng@htu.edu.cn; Green Catalysis Center and College of Chemistry, Zhengzhou University Zhengzhou Henan 450001 China; College of Chemistry and Chemical Engineering, Mudanjiang Normal University Mudanjiang Heilongjiang 157011 China

## Abstract

A general approach for the synthesis of covalent and ionic amine borane complexes containing trinitromethyl fragments has been developed through metathesis reactions between amine chloroborane complexes and potassium salt of trinitromethyl (K[C(NO_2_)_3_]). Five covalent and ionic trinitromethyl amine borane complexes have been synthesized in good yields with high purity and it is found that the ionic complex, [H_2_B(NH_3_)_2_][C(NO_2_)_3_], might be a promising energetic material on the basis of the investigation of its thermal decomposition behaviour.

## Abstract 2

A general approach has been developed through which five covalent and ionic amine borane complexes containing trinitromethyl fragments were synthesized.

## Graphical abstract

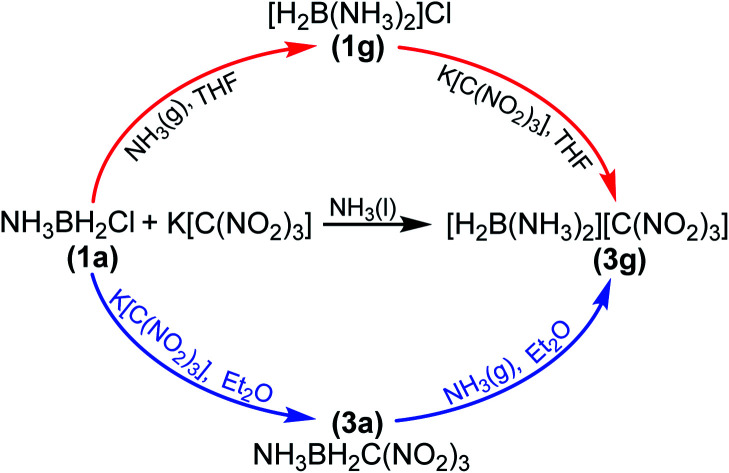

## Introduction

Nitroform (CH(NO_2_)_3_) holds a unique position among nitro compounds as it is a valuable starting material for the preparation of propellant and explosive components due to its high oxygen content.^1^ Ioffe group reported the syntheses and transformations of trinitromethylborane complexes with cyclic ethers and aromatic N-containing heterocycles, and also discussed the principle of the reactions.^2^ However, they attempted to obtain *N*,*N*-dinitroamidoborane complexes using the same method, but failed.^2*a*^ Klapötke and coworkers studied the reactions of boron oxide (B_2_O_3_) with various nitro-substituted ethanols (2-nitroethanol, 2-fluoro-2,2-dinitroethanol, and 2,2,2-trinitroethanol) to furnish the corresponding nitroethyl borates B(OCH_2_CH_2_NO_2_)_3_, B(OCH_2_CF(NO_2_)_2_)_3_, and B(OCH_2_C(NO_2_)_3_)_3_.^3^ The compound B(OCH_2_C(NO_2_)_3_)_3_ can be used as green-light-emitting pyrotechnic composition.^4^

Interestingly, C(NO_2_)_3_, as an oxidizing group, can be introduced into the design of high-energy molecules to co-exist with powerful reducing borohydride in a single covalently bonded structure.^5^ In 2013, Christe group used CH(NO_2_)_3_ and NaBH_4_ as starting materials to synthesize [Na(glyme)_2_][BH_3_C(NO_2_)_3_].^5^ Subsequently, they employed metathesis reactions to convert this salt into PNP^+^ and PPh^4+^ analogs that were stable for several months at room temperature. In 2015, ammonia-dinitro-amidoborane, NH_3_BH_2_N(NO_2_)_2_ was synthesized by the reaction of dinitroamine (HN(NO_2_)_2_) with ammonia borane (NH_3_BH_3_). This compound is expected to have a good performance as an explosive being comparable to that of pentaerythritol tetranitrate and significantly greater than that of trinitrotoluene.^6^ So, highly energetic oxidized analogs have attracted attention recently.

NH_3_BH_3_, a potential hydrogen storage material, has received immense interest in the past twenty years owing to its high percentage of hydrogen (19.6 wt%), excellent stability at room temperature, and release of hydrogen under mild conditions.^7^ It can also provide both a proton and hydride in chemical reactions under mild conditions.^8^ In NH_3_BH_3_, the nitrogen atom can be bonded to proton, hydrocarbon, hydroxyl, oxygen and other groups,^9^ and the boron atom can be bonded to hydride, hydrocarbon, oxygen, oxynitride, halogen and other electron donors that can interact with the empty orbital on boron.^10^ Many derivatives could be obtained from NH_3_BH_3_, including ammonia monochloroborane (NH_3_BH_2_Cl)^11–14^ which is an important intermediate for the synthesis of a series of more complex boron compounds^15^ such as R_1_R_2_N=BH_2_ (ref. 12*a*) and amorphous boron nitride.^11*b*^

In this work, ammonia and amine monochloroborane complexes (ABH_2_Cl, A = NH_3_, aliphatic primary, secondary, tertiary amine, and diamines, 1) were treated with K[C(NO_2_)_3_], a milder oxidizing reagent in comparison with CH(NO_2_)_3_,^16^ to prepare target product 3, with the general formula of ABH_2_C(NO_2_)_3_, containing both reducing and oxidizing fragments in a single covalently bonded structure. Furthermore, an ionic complex, [H_2_B(NH_3_)_2_][C(NO_2_)_3_], was synthesized from 3a.

## Results and discussion

**Scheme 1:**
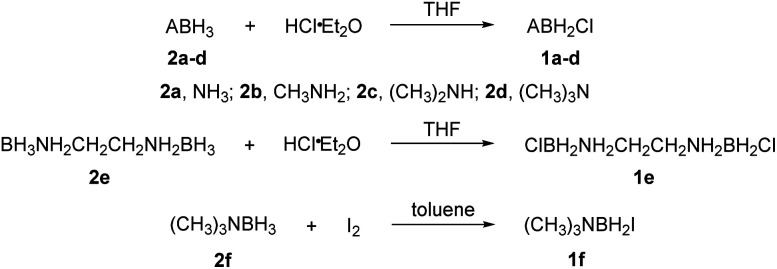
Synthesis of compounds 1a–f.

**Scheme 2:**
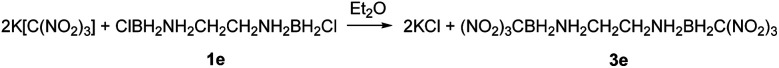
Synthesis of compound 3e.

**Scheme 3:**
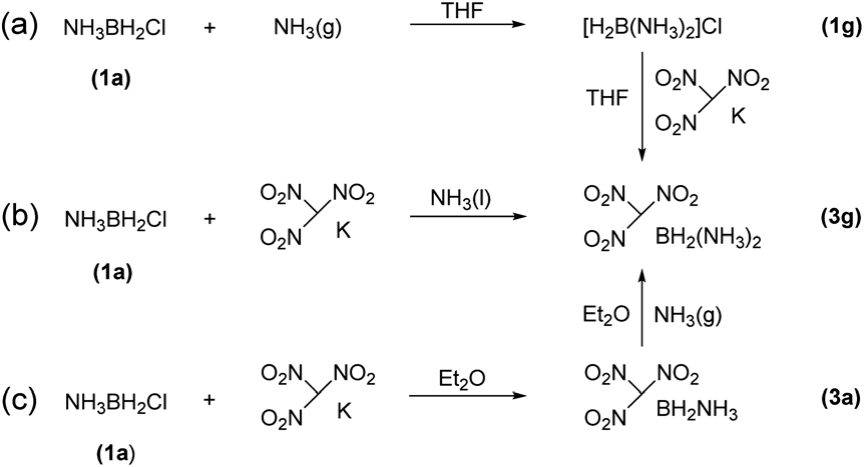
The methods for the synthesis of 3g.

The reactions of each amine borane complex ABH_3_ (2a–d) with HCl diethyl ether solution at room temperature resulted in the formation of amine monochloroborane complexes (1a–d) in good yields. Ethylenediaminebisborane (2e) reacted with HCl·Et_2_O at 1 : 2 ratio to form ClBH_2_NH_2_CH_2_CH_2_NH_2_BH_2_Cl (1e). Trimethylamine borane (2f) reacted with iodine to afford trimethylamine monoiodoborane complex (1f) (Scheme 1). These amine halogenated borane complexes (1a–f) further reacted with K[C(NO_2_)_3_] to produce the products (Table 1, 3a–c) by metathesis reactions. Compound (3e) (Table 1) was also synthesized by the reaction of 1e with K[C(NO_2_)_3_] according to Scheme 2. It should be noted that ammonia monochloroborane (1a) can further react with ammonia gas in THF to afford [H_2_B(NH_3_)_2_]Cl (1g) ((a) in Scheme 3). 1a is a covalent complex in which the Cl–B bond is a typical covalent bond, in accordance with amine monochloroborane complexes 1a–d. However, 1g shows a ionic behaviour, similar to the diammoniate of diborane ([H_2_B(NH_3_)_2_][BH_4_]).^17^ The reaction of 1g and K[C(NO_2_)_3_] leads to the formation of an ionic compound, 3g (Scheme 3). On the other hand, attempts for the syntheses of (CH_3_)_3_NBH_2_C(NO_2_)_3_ have failed, ether by chloroborane or iodoborane intermediates, probably due to the steric hindrance of the trimethylamine group. In general, five amine borane containing the trinitromethyl group, four covalent and one ionic complexes (Table 1, 3a–c, 3e, 3g), were successfully synthesized in good yields with high purity.

**Table 1:** Synthesis of 3a–g from 1a–g and K[C(NO_2_)_3_]

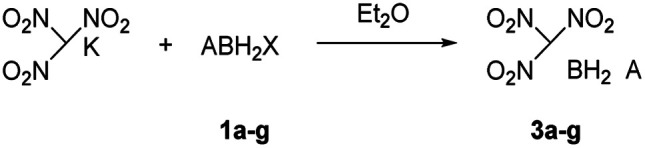				
Entry	Comps.	A	X (1)	Yields of 3^a^ (%)
1	a	NH_3_	Cl	65
2	b	CH_3_NH_2_	Cl	62
3	c	(CH_3_)_2_NH	Cl	67
4	d	(CH_3_)_3_N	Cl	No reaction
5	e	(CH_2_NH_2_)_2_	Cl	62
6	f	(CH_3_)_3_N	I	No reaction
7	g	(NH_3_)_2_	Cl	65^b^

a Isolated yield.

b Yield of synthesis method a.

It is worthy to note that 3g was firstly synthesized by Ioffe group in 2014 (3a in Scheme 3).^2*b*^ We provide two alternative routes in this work. Scheme 3a shows the Ioffe group's method as described above,^2*b*^1a reacted with excess NH_3_ to convert into 1g and then reacted with K[C(NO_2_)_3_] to form a yellow residue 3g in a yield of 65%. In our work, as shown in Scheme 3b, K[C(NO_2_)_3_] directly reacted with 1a in liquid NH_3_ at −78 °C to give yellow product 3g with a yield of 68%; and Scheme 3c, K[C(NO_2_)_3_] reacted with 1a in ethyl ether first and then with excess NH_3_ gas to give 3g, the yield was 59%. In comparison, pathway (b) is a one-step procedure with high efficiency. Further study shows that 3g is an ionic complex, it has good solubility in organic solvents, such as tetrahydrofuran (THF) and acetonitrile (CH_3_CN), different from (NH_3_)_2_BH_2_Cl.^14^3g also showed good stability towards air and moisture even for several months at room temperature. Thus, it has wide potential applications as a high-energy-density material.

The ^11^B NMR data of ABH_2_X (X = Cl, I, and C(NO_2_)_3_) are summarized in Table 2. The chemical shift of the B atom in ABH_2_C(NO_2_)_3_ shifts downfield about 4 ppm in comparison with that of the B atom in the corresponding ABH_2_Cl complexes. This can be attributed the strong electron-withdrawing ability of the C(NO_2_)_3_ group relative to Cl. With increasing each one methyl group bonded in the N atom, on the other hand, the chemical shift of the B signal shifts downfield about 2 ppm (Table 2) in either ABH_2_Cl or ABH_2_C(NO_2_)_3_ complexes. These change trends are consistent with those of the chemical shift of the B atom in amine boranes. For the amine chloroborane complexes or trinitromethylamidoborane complexes, whether the compound is mono-substituted (1b and 3b) or bis-substituted (1e and 3e), little effect was observed on the chemical shift of ABH_2_X (Table 2, entries 2 and 5). The B signal of [H_2_B(NH_3_)_2_]Cl and [H_2_B(NH_3_)_2_][C(NO_2_)_3_] are almost identical (Table 2, entry 7) because they are ionic compounds so that the effect of the different counter-anion on the chemical shift of the B atom in the [H_2_B(NH_3_)_2_]^+^ cation is weak. In contrast, the effect is more pronounced in covalent complexes (1a–f and 3a–c, e) as described above because of the direct N–B and B–C bonding. In addition, the chemical shift of the proton of the BH_2_ group in ABH_2_C(NO_2_)_3_ in ^1^H NMR are also summarized in Table S1,† all proton signals appeared at about *δ* 2 ppm, similar to those of ABH_2_Cl.^11–14^ This indicated that the change of substituents may not influence the chemical shift of the BH_2_ group in ABH_2_C(NO_2_)_3_.

**Table 2:** ^11^B NMR of 1 and 3^a^

Entry	Comps.	A	X (1)	^11^B NMR (ppm)	X (3)	^11^B NMR (ppm)
1	a	NH_3_	Cl	−8.73	C(NO_2_)_3_	−4.8
2	b	CH_3_NH_2_	Cl	−6.36	C(NO_2_)_3_	−2.32
3	c	(CH_3_)_2_NH	Cl	−3.47	C(NO_2_)_3_	−0.22
4	d	(CH_3_)_3_N	Cl	0.21	C(NO_2_)_3_	—
5	e	(NH_2_CH_2_)_2_	Cl	−6.47^b^	C(NO_2_)_3_	−2.93^c^
6	f	(CH_3_)_3_N	I	−10.57	C(NO_2_)_3_	—
7	g	(NH_3_)_2_	Cl	−13.54	C(NO_2_)_3_	−13.06

a A is the lewis base, X is the substituent group.

b Molecular formula (NH_2_CH_2_BH_2_Cl)_2_.

c Molecular formula [NH_2_CH_2_BH_2_C(NO_2_)_3_]_2_.

**Fig. 1:**
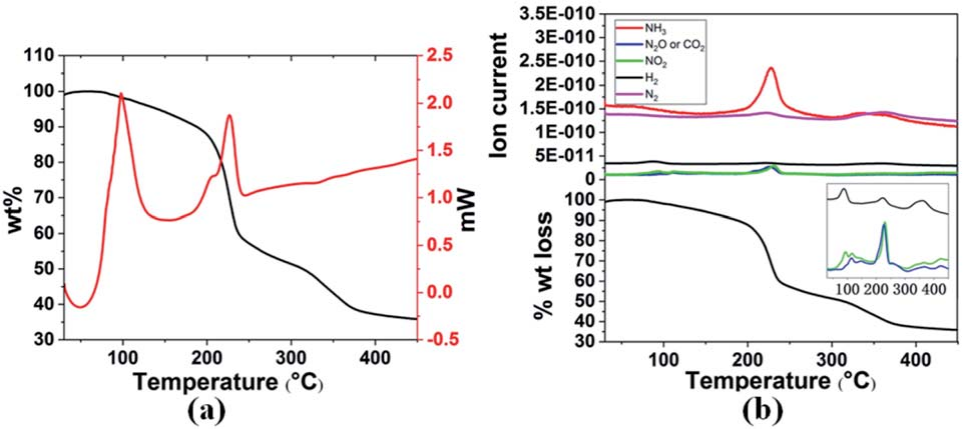
(a) TGA and DSC curves and (b) TGA-MS analysis of 3a in the temperature range 30–400 °C with a heating rate of 3 °C min^−1^.

**Fig. 2:**
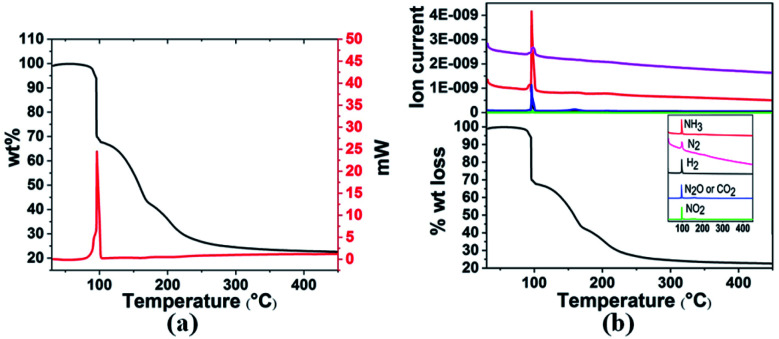
(a) TGA and DSC curves (b) TGA-MS analysis of 3g in the temperature range 30–400 °C with a heating rate of 3 °C min^−1^.

Thermal decomposition of 3a and 3g was studied by DSC and TGA-MS. As shown in Fig. 1 and 2, thermal decomposition resulted in the generation of H_2_, N_2_, NH_3_, CO_2_, N_2_O and NO_2_, hence the decomposition is believed to proceed according to eqn (1) and (2), respectively.

Gaseous products were analysed by mass spectrometry (MS), and solid residues were determined by IR and XRD. It was observed that 3a starts decomposing at 98 °C and the first-stage weight loss is only 1.7 wt%, corresponding to the evolution of hydrogen and nitrogen dioxide, and the *m*/*z* 44 signal is assigned to N_2_O or CO_2_ evolution. The decomposition behaviour of 3a is similar to that of the energetic oxidizer hydrazinium nitroformate (N_2_H_5_C(NO_2_)_3_, HNF), as shown in eqn (3).^18^ The second-stage weight loss is as large as 31.2 wt%, associated with H_2_, N_2_, NH_3_, CO_2_, N_2_O and NO_2_ evolution. Both the first and second steps for 3a are exothermic events, the *m*/*z* 2, 28, 17, 44, and 46 signals recorded by the MS during the TGA-MS experiment to 400 °C are overlaid in Fig. 1b. At higher temperatures, it further decomposes and boron oxide was formed (Fig. S1 and S2†).

1 2NH_3_BH_2_C(NO_2_)_3_ → 2NH_3_ + 2H_2_ + 2NO_2_ + N_2_O + 2CO_2_ + B_2_O_3_ + N_2_

2 2[H_2_B(NH_3_)_2_][C(NO_2_)_3_] → 4NH_3_ + 2H_2_ + 2NO_2_ + N_2_O + 2CO_2_ + B_2_O_3_ + N_2_

3 2N_2_H_5_C(NO_2_)_3_ → NH_4_C(NO_2_)_3_ + N_2_O + 2H_2_O + H_2_CO

The thermal decomposition pattern of 3g is different from 3a. The large weight loss of 30.6 wt%, observed at 95 °C with strongly exothermic, is associated with H_2_, N_2_, NH_3_, CO_2_, N_2_O and NO_2_ evolution. At higher temperature, it further decomposes to release N_2_O, CO_2_ and NO_2_, resulting in boron oxide (Fig. S3 and S4†). The thermal decomposition behaviours of 3a and 3g are different from those of NH_3_BH_2_Cl, their parent compound. It was recorded that only H_2_ was released at the initial stage (eqn (4)), and then the second large weight loss was associated with both H_2_ and HCl (eqn (5)). At higher temperature, it further decomposes to evolve HCl and H_2_ to form boron nitride (eqn (6)).^11*c*^

4 NH_3_BH_2_Cl → H_2_ + (NH_2_BHCl)_*x*_

5
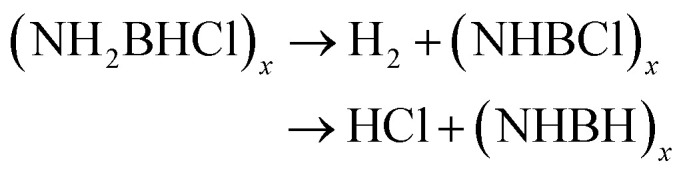
6
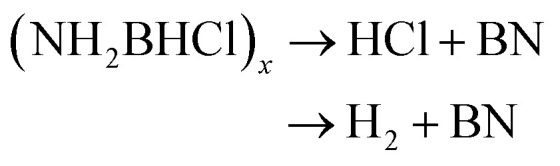


## Conclusions

In summary, we have developed general methods for the syntheses of both covalent and ionic complexes containing the amine borane reducing group and the trinitromethyl oxidizing group in one molecule under mild conditions. These complexes were successfully isolated in high yields and characterized by NMR and IR. Thermal decomposition was investigated by TGA-MS and DSC, and results implicated that compound 3g may be a promising explosive material. Further studies on the explosive property of 3g are in progress in our lab.

## Experimental

**CAUTION!** All nitrogen- and oxygen-rich compounds are potentially explosive energetic materials which should be handled with great care, although no hazards were observed during preparation and handling of these compounds. In any case, it is necessary to take proper precautions by employing all standard energetic materials safety procedures in experiments involving such substances, such as face shields, a leather apron, gloves, and hearing protection should be employed.

### General information

All manipulations were carried out under a nitrogen atmosphere using standard Schlenk techniques and glove box. The ^11^B NMR and ^11^B{^1^H} NMR spectra were recorded at 128 or 193 MHz spectrometers and externally referenced to BF_3_·OEt_2_ in C_6_D_6_ (*δ* = 0.00 ppm). The ^1^H NMR and ^1^H{^11^B} NMR spectra were obtained at 600 MHz spectrometer. The ^13^C NMR spectra were recorded at 151 MHz. IR spectra were measured by a Spectrum 400F. X-ray diffraction (XRD) data were obtained with a Rigaku D/max 2500 diffractometer using Cu/Kα radiation, *λ* = 0.1542 nm, 40 kV, 100 mA. The thermal behaviours of compounds 3a and 3g were determined by synchronous thermal analyses (TGA-DSC, Netzsch 449C Jupiter/QMS 403D). The samples were heated to 500 °C with a heating rate of 3 °C min^−1^, under a flowing Ar atmosphere.

Anhydrous nitric acid, sulphuric acid, acetic anhydride, KOH, anhydrous sodium sulfate, ethanol, and iodine were purchased from Sinopharm Chemical Reagents Co., Ltd. NH_3_BH_3_, MeNH_2_BH_3_, Me_2_NHBH_3_, Me_3_NBH_3_, BH_3_NH_2_CH_2_CH_2_NH_2_BH_3_ and HCl diethyl ether solution (1 mol L^−1^) were purchased from United Boron (Zhengzhou) Energy Materials S&T LLC and used as received. Tetrahydrofuran (THF), diethyl ether (Et_2_O), *n*-hexane, and toluene were dried over sodium and freshly distilled prior to use. *N*,*N*-Dimethylformamide (DMF) was dried by molecular sieves.

Amine chloroborane (1a–d) and K[C(NO_2_)_3_] were prepared according to the literature methods.^11,19^

#### 1a

Yield: 92%; ^11^B NMR (128 MHz, THF): *δ* −8.73 (t, *J*_B–H_ = 114.3 Hz) ppm (Fig. S5a†). ^11^B{^1^H} NMR (128 MHz, THF): *δ* −8.73 (s) ppm (Fig. S5b†).

#### 1b

Yield: 90%; ^11^B NMR (193 MHz, THF): *δ* −6.36 (t, *J*_B–H_ = 120.2 Hz) ppm (Fig. S6a†). ^11^B{^1^H} NMR (193 MHz, THF): *δ* −6.36 (s) ppm (Fig. S6b†).

#### 1c

Yield: 86%; ^11^B NMR (193 MHz, THF): *δ* −3.47 (t, *J*_B–H_ = 120.6 Hz) ppm (Fig. S7a†). ^11^B{^1^H} NMR (193 MHz, THF): *δ* −3.47 (s) ppm (Fig. S7b†).

#### 1d

Yield: 90%; ^11^B NMR (193 MHz, THF): *δ* 0.21 (t, *J*_B–H_ = 123.0 Hz) ppm (Fig. S8a†). ^11^B{^1^H} NMR (193 MHz, THF): *δ* 0.21(s) ppm (Fig. S8b†).

#### K[C(NO_2_)_3_]

Yield of 80%; IR (cm^−1^): 1589 (s), 1363 (s), 1301 (s), 823 (m), (Fig. S31†).

### Synthesis of ClBH_2_NH_2_CH_2_CH_2_NH_2_BH_2_Cl (1e)

To a solution of ethylenediaminebisborane (0.088 g, 1 mmol) in THF (2 mL) was added HCl diethyl ether solution (2.2 mmol, 2.2 mL) *via* syringe at ambient temperature. The reaction was monitored by ^11^B NMR and after about 30 min the reaction was finished. Then the mixture was filtered and solvent was removed from the filtrate under a dynamic vacuum to leave a white product (0.144 g, yield 92%). ^11^B NMR (193 MHz, CD_3_CN): *δ* −6.47 (t, *J*_B–H_ = 107.7 Hz) ppm (Fig. S9a†). ^11^B{^1^H} NMR (193 MHz, THF): *δ* −6.47 (s) ppm (Fig. S9b†).

### Synthesis of (CH_3_)_3_NBH_2_I (1f)

To a solution of trimethylamine borane (0.073 g, 1 mmol) in toluene (2 mL) was added I_2_ (0.127 g, 0.5 mmol) in toluene (5 mL) *via* syringe at ambient temperature. The reaction was monitored by ^11^B NMR and after about 1 h the reaction was finished. After reaction, the mixture was filtered and solvent was removed from the filtrate under a dynamic vacuum to leave a white product (0.169 g, yield 85%). ^11^B NMR (128 MHz, toluene): *δ* −10.57 (t, *J*_B–H_ = 130.9 Hz) ppm (Fig. S10a†). ^11^B{^1^H} NMR (128 MHz, toluene): *δ* −10.57 (s) ppm (Fig. S10b†).

### Synthesis of [(NH_3_)_2_BH_2_]Cl (1g)

Ammonia chloroborane (0.073 g, 1 mmol) was placed in a 10 mL flask, and the 2 mL of THF was injected into the flask. Then excess NH_3_ was bubbled into the flask at room temperature and white precipitate was formed immediately. After filtration, THF was removed from the filtrate under dynamic vacuum to leave a white powder product (0.078 g, yield 94%). ^11^B NMR (193 MHz, DMF): *δ* −13.54 (t, *J*_B–H_ = 104.4 Hz) ppm (Fig. S11a†). ^11^B{^1^H} NMR (193 MHz, DMF): *δ* −13.54 (s) ppm (Fig. S11b†).

### General procedure for the synthesis of ABH_2_C(NO_2_)_3_ (3a–c)

To a solution of aminoborane (1 mmol) (2a: 0.031 g; 2b: 0.045 g; 2c: 0.059 g) in THF (2 mL) was added HCl diethyl ether (1.1 mmol, 1.1 mL) *via* syringe at ambient temperature. The reaction was monitored by ^11^B NMR and after about 30 min the reaction was finished. Then the mixture was filtered and solvent was removed from the filtrate under a dynamic vacuum to leave the products of 1a–c. The prepared 1a–c and K[C(NO_2_)_3_] (0.208 g, 1.1 mmol) were added to the flask and then added 5 mL diethyl ether at ambient temperature. The yellow solid product was separated by filtration using a filter cannula and dried *in vacuo*.

#### 3a

Yield: 65% (0.117 g); ^11^B NMR (193 MHz, CD_3_CN) *δ* −4.8 (t, *J*_B–H_ = 115.9 Hz) (Fig. S12a†). ^11^B{^1^H} NMR (193 MHz, CD_3_CN) *δ* −4.8 (s) (Fig. S12b†). ^1^H NMR (600 MHz, CD_3_CN) *δ* 4.4 (t, *J*_N–H_ = 45.8 Hz, 3H of NH_3_), 2.89–2.31 (m, 2H of BH_2_) (Fig. S13a†). ^1^H{^11^B} NMR (600 MHz, CD_3_CN) *δ* 4.4 (t, *J*_N–H_ = 45.8 Hz, 3H of NH_3_), 2.61 (s, 2H of BH_2_) (Fig. S13b†). IR (cm^−1^): 3326 (s), 3217 (s), 2448 (w), 1566 (m), 1514 (s), 1411 (s), 1279 (s), 1176 (s), 794 (m), 734 (w) (Fig. S14†).

#### 3b

Yield: 62% (0.120 g); ^11^B NMR (193 MHz, CD_3_CN) *δ* −2.32 (t, *J*_B–H_ = 116.5 Hz) (Fig. S15a†). ^11^B{^1^H} NMR (193 MHz, CD_3_CN) *δ* −2.32 (s) (Fig. S15b†). ^1^H NMR (600 MHz, CD_3_CN) *δ* 4.68 (t, *J*_N–H_ = 44.0 Hz, 2H of NH_2_), 2.95–2.13 (m, 2H of BH_2_), 2.35 (t, *J*_C–H_ = 5.8 Hz, 3H of CH_3_) (Fig. S16a† ). ^1^H{^11^B} NMR (600 MHz, CD_3_CN) *δ* 4.68 (t, *J*_N–H_ = 43.0 Hz, 2H of NH_2_), 2.53 (s, 2H of BH_2_), 2.35 (t, *J*_C–H_ = 5.8 Hz, 3H of CH_3_) (Fig. S16b†). ^13^C NMR (151 MHz, CD_3_CN) *δ* 29.48 (Fig. S17†). IR (cm^−1^): 3438 (w), 3093 (w), 1514 (s), 1422 (s), 1384 (s), 1279 (s), 1177 (s), 927 (w), 794 (m), 734 (m) (Fig. S18†).

#### 3c

Yield: 67% (0.139 g); ^11^B NMR (193 MHz, CD_3_CN) *δ* 0.22 (t, *J*_B–H_ = 117.1 Hz) (Fig. S19a†). ^11^B{H} NMR (193 MHz, CD_3_CN) *δ* 0.22 (s) (Fig. S19b†). ^1^H NMR (600 MHz, CD_3_CN) *δ* 4.92 (s, ^1^H of NH), 2.48 (d, *J*_C–H_ = 5.7 Hz, 6H of CH_3_), 2.90–2.09 (m, 2H of BH_2_) (Fig. S20a†). ^1^H{^11^B} NMR (600 MHz, CD_3_CN) *δ* 4.92 (s, ^1^H of NH), 2.48 (d, *J*_C–H_ = 5.8 Hz, 6H of CH_3_), 2.47 (s, 2H of BH_2_) (Fig. S20b†). ^13^C NMR (151 MHz, CD_3_CN) *δ* 39.55 (Fig. S21†). IR (cm^−1^): 3441 (m), 3058 (m), 2779 (m), 2435 (w), 1496 (s), 1422 (s), 1384 (s), 1277 (s), 1161 (m), 1022 (w), 924 (w), 793 (m), 733 (m) (Fig. S22†).

### Synthesis of [CH_2_NH_2_BH_2_C(NO_2_)_3_]_2_ (3e)

To a solution of ethylenediaminebisborane (0.088 g, 1 mmol) in THF (2 mL) was added HCl diethyl ether solution (2.2 mmol, 2.2 mL) *via* syringe at ambient temperature. The reaction was monitored by ^11^B NMR and after about 30 min the reaction was finished. Then the solvent was removed from the filtrate under a dynamic vacuum to leave product. The prepared 1e and K[C(NO_2_)_3_] (0.416 g, 2.2 mmol) were added to the flask and then added 5 mL diethyl ether at ambient temperature. The yellow solid product was separated by filtration using a filter cannula and dried *in vacuo*.

#### 3e

Yield: 62% (0.239 g); ^11^B NMR (193 MHz, CD_3_CN) *δ* −2.93 (t, *J*_B–H_ = 116.6 Hz) (Fig. S23a†). ^11^B{H} NMR (193 MHz, CD_3_CN) *δ* −2.94 (s) (Fig. S23b†). ^1^H NMR (600 MHz, CD_3_CN) *δ* 4.91 (s, 2H of NH_2_), 2.98 (s, 3H of CH_3_), 2.90–2.18 (m, 2H of BH_2_) (Fig. S24a†). ^1^H{^11^B} NMR (600 MHz, CD_3_CN) *δ* 4.91 (s, 2H of NH_2_), 2.98 (s, 3H of CH_3_), 2.56 (s, 2H of BH_2_) (Fig. S24b†). ^13^C NMR (151 MHz, CD_3_CN) *δ* 42.28 (Fig. S25†). IR (cm^−1^): 3172 (w), 3057 (w), 1608 (w), 1519 (w), 1361 (m), 1296 (s), 1087 (m), 1032 (m), 918 (m), 822 (m), 778 (m), 692 (m), 461 (w) (Fig. S26†).

### Synthesis of [H_2_B(NH_3_)_2_][C(NO_2_)_3_] (3g)

To a solution of ammonia borane (0.031 g, 1 mmol) in THF (2 mL) was dropwise added HCl diethyl ether (1.1 mmol, 1.1 mL) *via* syringe at ambient temperature. The white solid product (1a) was separated by filtration using a filter cannula dried *in vacuo* for use.

(a) Repeated the literature method. The prepared 1a was placed in a flask, and the 2 mL of THF was injected into the flask. Then excess NH_3_ was bubbled into the flask for 30 min under stirring at room temperature and white precipitate was produced immediately. Then K[C(NO_2_)_3_] (0.208 g, 1.1 mmol) in THF (5 mL) was added into the flask, the reaction mixture was stirred for 2 h and the solution turned from colourless and transparent to yellow. After filtration to remove the formed KCl, THF was removed from the filtrate under dynamic vacuum to give a yellow powder product (3g, 0.129 g, yield 65%).

(b) The prepared 1a and K[C(NO_2_)_3_] (0.208 g, 1.1 mmol) were added to a flask and then 5 mL of liquid NH_3_ was condensed into the flask at −78 °C and stirred for 2 hours. Then the reaction was warm up to room temperature and liquid NH_3_ was volatilized completely to leave white and yellow powder precipitate (KCl and 3g). The yellow precipitate was extracted with 20 mL of THF. Removal of THF from the filtrate under dynamic vacuum gave a yellow powder product (0.133 g, yield 68%).

(c) The prepared 1a and K[C(NO_2_)_3_] (0.208 g, 1.1 mmol) were added to a flask and the 5 mL of ethyl ether was injected into the flask. The reaction was stirred for 2 h, then excess NH_3_ was bubbled into the flask and white precipitate was produced immediately, the solution turned to yellow. Removal of ethyl ether from the filtrate under dynamic vacuum gave a yellow product (3g, 0.117 g, yield 59%).

#### 3g

^11^B NMR (193 MHz, CD_3_CN) *δ* −13.06 (t, *J*_B–H_ = 110.9 Hz) (Fig. S27a†). ^11^B^1^{H} NMR (193 MHz, CD_3_CN) *δ* −13.05 (s) (Fig. S27b†). ^1^H NMR (600 MHz, CD_3_CN) *δ* 4.40 (t, *J*_N–H_ = 47.4 Hz, 6H of NH_3_), 2.40–1.56 (m, 2H of BH_2_) (Fig. S28a†).^1^H{^11^B} NMR (600 MHz, CD_3_CN) *δ* 4.40 (t, *J*_N–H_ = 47.6 Hz, 6H of NH_3_), 2.02 (m, 2H of BH_2_) (Fig. S28b†). IR (cm^−1^): 3274 (m), 2444 (w), 2409 (w), 2338 (w), 1514 (s), 1408 (s), 1384 (s), 1273 (s), 1173 (m), 1093 (w), 1028 (w), 869 (w), 792 (s), 734 (s), 693 (w) (Fig. S29†).

## Conflicts of interest

There are no conflicts to declare.

## Supplementary Material

RA-011-D1RA00440A-s001
