# Supplementary material for: A general method for the synthesis of covalent and ionic amine borane complexes containing trinitromethyl fragments
Source: RSC Adv. 2021 Mar 5;11(16):9740–5. doi: 10.1039/d1ra00440a (PMC8695509; doi:10.1039/d1ra00440a)
Supplement: RA-011-D1RA00440A-s001 [file RA-011-D1RA00440A-s001.pdf]

Electronic Supplementary Material (ESI) for RSC Advances.
This journal is © The Royal Society of Chemistry 2021

Electronic Supplementary Information for:

A General Method for the Synthesis of Covalent and Ionic Amine Borane Complexes Containing Trinitromethyl Fragments

Jin Wang,[a,c] Ming-Yue Ju,[a] Xi-Meng Chen,*[a] and Xuenian Chen*[a,b]

[a] *School of Chemistry and Chemical Engineering, Henan Key Laboratory of Boron Chemistry and Advanced Energy Materials, Henan Normal University, Xinxiang, Henan 453007, China*

[b] *Green Catalysis Center and College of Chemistry, Zhengzhou University, Zhengzhou, Henan 450001, China*

[c] *College of Chemistry and Chemical Engineering, Mudanjiang Normal University, Mudanjiang, Heilongjiang 157011, China*

E-mails: xnchen@htu.edu.cn; chenximeng@htu.edu.cn

Table S1. The chemical shift of hydrides in BH_2 group in 3a-3g in ^1H NMR[a]. 3

Fig. S1. IR spectrum of the residue from the TGA−MS experiment of $NH_3BH_2C(NO_2)_3$ (3a) (The spectrum is similar to that of B_2O_3)...3

Fig. S2. X-ray powder diffraction pattern of the residue from the TGA−MS experiment of $NH_3BH_2C(NO_2)_3$ (3a) (The peaks are related to B_2O_3) 4

Fig. S3. IR spectrum of the residue from the TGA−MS experiment of $[H_2B(NH_3)_2][C(NO_2)_3]$ (3g) (The spectrum is similar to that of B_2O_3). 4

Fig. S4. X-ray powder diffraction pattern of the residue from the TGA−MS experiment of $[H_2B(NH_3)_2][C(NO_2)_3]$ (3g) (The peaks are related to B_2O_3). 5

Fig. S5. ^{11}B (a) and $^{11}B\{^1H\}$ NMR (b) spectra of NH_3BH_2Cl (1a) in THF. 5

Fig. S6. ^{11}B (a) and $^{11}B\{^1H\}$ NMR (b) spectra of $CH_3NH_2BH_2Cl$ (1b) in THF. 6

Fig. S7. ^{11}B (a) and $^{11}B\{^1H\}$ NMR (b) spectra of $(CH_3)_2NHBH_2Cl$ (1c) in THF........................6

Fig. S8. ^{11}B (a) and $^{11}B\{^1H\}$ NMR (b) spectra of $(CH_3)_3NBH_2Cl$ (1d) in THF. 7

Fig. S9. ^{11}B (a) and $^{11}B\{^1H\}$ NMR (b) spectra of $ClBH_2NH_2CH_2CH_2NH_2BH_2Cl$ (1e) in THF........7

Fig. S10. ^{11}B (a) and $^{11}B\{^1H\}$ NMR (b) spectra of $(CH_3)_3NBH_2I$ (1f) in toluene. 7

Fig. S11. ^{11}B (a) and $^{11}B\{^1H\}$ NMR (b) spectra of $(NH_3)_2BH_2Cl$ (1g) in DMF. 8

Fig. S12. ^{11}B (a) and $^{11}B\{^1H\}$ NMR (b) spectra of $NH_3BH_2C(NO_2)_3$ (3a) in CD_3CN. 8

Fig. S13. ^{1}H (a) and $^{1}H\{^{11}B\}$ NMR (b) spectra of $NH_3BH_2C(NO_2)_3$ (3a) in CD_3CN. 8
Fig. S14. IR spectrum of $NH_3BH_2C(NO_2)_3$ (3a). 9
Fig. S15. ^{11}B (a) and $^{11}B\{^{1}H\}$ NMR (b) spectra of $CH_3NH_2BH_2C(NO_2)_3$ (3b) in CD_3CN............9
Fig. S16. ^{1}H (a) and $^{1}H\{^{11}B\}$ NMR (b) spectra of $CH_3NH_2BH_2C(NO_2)_3$ (3b) in CD_3CN.............9
Fig. S17. ^{13}C NMR spectrum of $CH_3NH_2BH_2C(NO_2)_3$ (3b) in CD_3CN....................................10
Fig. S18. IR spectrum of $CH_3NH_2BH_2C(NO_2)_3$ (3b). 10
Fig. S19. ^{11}B (a) and $^{11}B\{^{1}H\}$ NMR (b) spectra of $(CH_3)_2NHBH_2C(NO_2)_3$ (3c) in CD_3CN.........10
Fig. S20. ^{1}H (a) and $^{1}H\{^{11}B\}$ NMR (b) spectra of $(CH_3)_2NHBH_2C(NO_2)_3$ (3c) in CD_3CN. 11
Fig. S21. ^{13}C NMR spectrum of $(CH_3)_2NHBH_2C(NO_2)_3$ (3c) in CD_3CN..................................11
Fig. S22. IR spectrum of $(CH_3)_2NHBH_2C(NO_2)_3$ (3c)...12
Fig. S23. ^{11}B (a) and $^{11}B\{^{1}H\}$ NMR (b) spectra of $[CH_2NH_2BH_2C(NO_2)_3]_2$ (3e) in CD_3CN. 12
Fig. S24. ^{1}H (a) and $^{1}H\{^{11}B\}$ NMR (b) spectra of $[CH_2NH_2BH_2C(NO_2)_3]_2$ (3e) in CD_3CN. 12
Fig. S25. ^{13}C NMR spectrum of $[CH_2NH_2BH_2C(NO_2)_3]_2$ (3e) in CD_3CN. 13
Fig. S26. IR spectrum of $[CH_2NH_2BH_2C(NO_2)_3]_2$ (3e). 13
Fig. S27. ^{11}B (a) and $^{11}B\{^{1}H\}$ NMR (b) spectra of $[H_2B(NH_3)_2][C(NO_2)_3]$ (3g) in CD_3CN. 14
Fig. S28. ^{1}H (a) and $^{1}H\{^{11}B\}$ NMR (b) spectra of $[H_2B(NH_3)_2][C(NO_2)_3]$ (3g) in CD_3CN. 14
Fig. S29. IR spectrum of $[H_2B(NH_3)_2][C(NO_2)_3]$ (3g)...15
Fig. S30. X-ray powder diffraction pattern of KCl. 15
Fig. S31. IR spectrum of $K[C(NO_2)_3]$. 16

Table S1. The chemical shift of hydrides in BH_2 group in 3a-3g in ^1H NMR[a].

Entry	Comps.	A	^1H NMR (ppm)
1	**3a**	NH_3	2.61
2	**3b**	CH_3NH_2	2.53
3	**3c**	$(CH_3)_2NH$	2.47
4	**3d**	$(CH_3)_3N$	-
5	**3e**	$(NH_2CH_2)_2$	2.02[b]
6	**3f**	$(CH_3)_3N$	-
7	**3g**	$(NH_3)_2$	2.56

[a] "A" is a Lewis base; [b] molecular formula $[NH_2CH_2BH_2C(NO_2)_3]_2$.

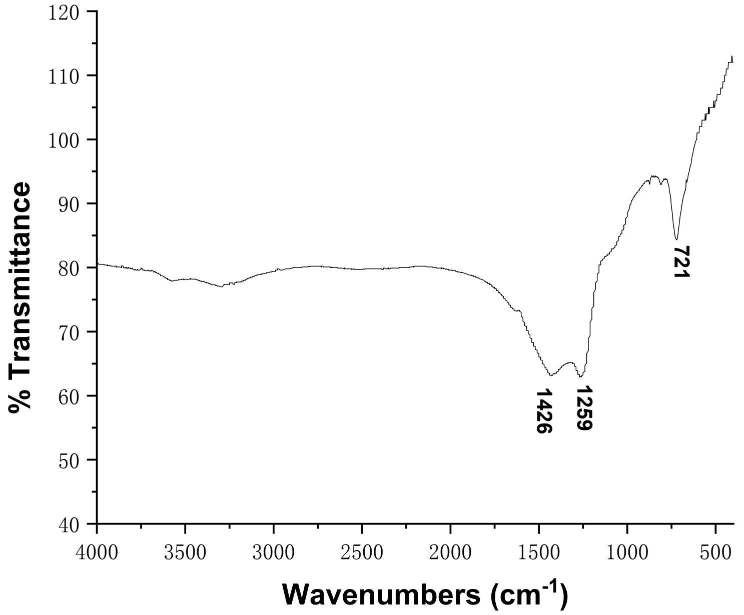

Fig. S1. IR spectrum of the residue from the TGA−MS experiment of $NH_3BH_2C(NO_2)_3$ (3a) (The spectrum is similar to that of B_2O_3).

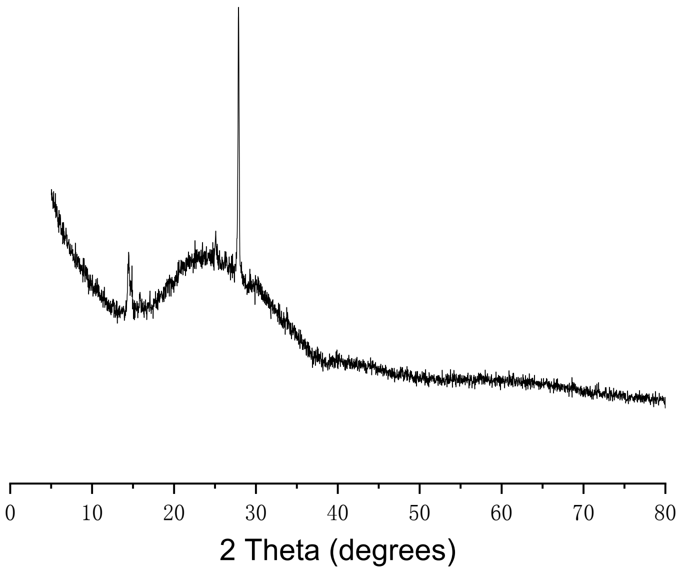

Fig. S2. X-ray powder diffraction pattern of the residue from the TGA−MS experiment of $NH_3BH_2C(NO_2)_3$ (3a)（The peaks are related to B_2O_3）.

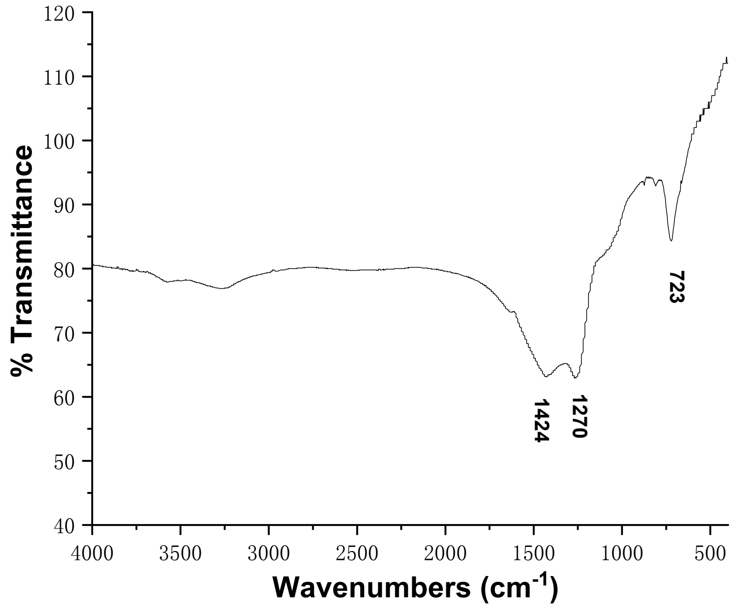

Fig. S3. IR spectrum of the residue from the TGA−MS experiment of $[H_2B(NH_3)_2][C(NO_2)_3]$ (3g) (The spectrum is similar to that of B_2O_3).

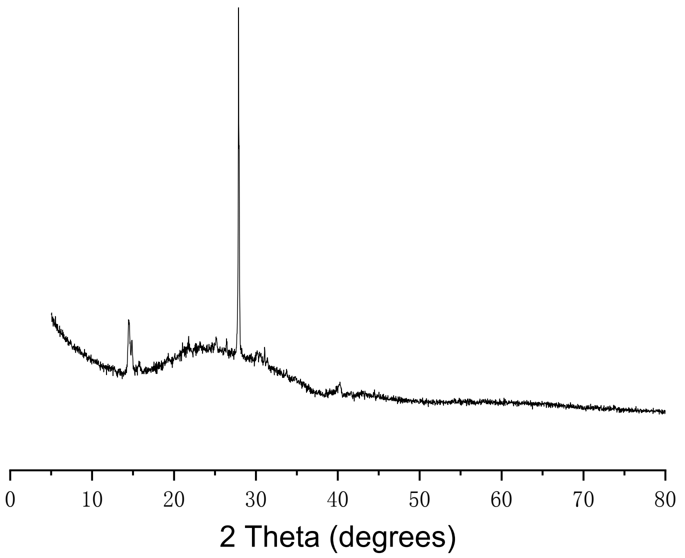

Fig. S4. X-ray powder diffraction pattern of the residue from the TGA−MS experiment of $[H_2B(NH_3)_2][C(NO_2)_3]$ (3g) (The peaks are related to B_2O_3).

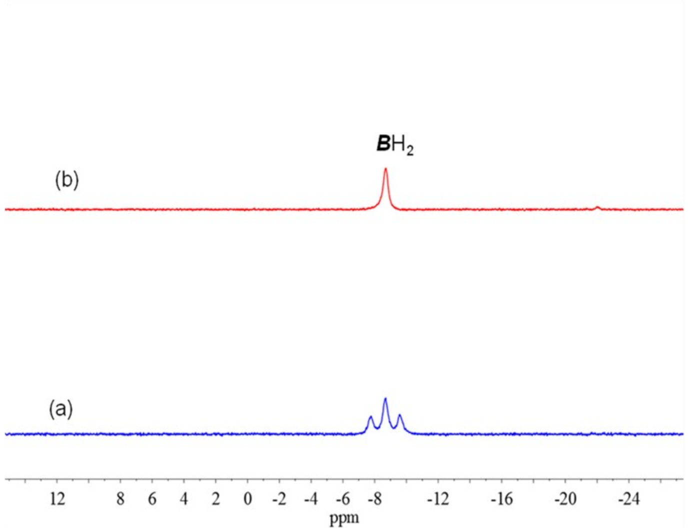

Fig. S5. ^{11}B (a) and $^{11}B\{^1H\}$ NMR (b) spectra of NH_3BH_2Cl (1a) in THF.

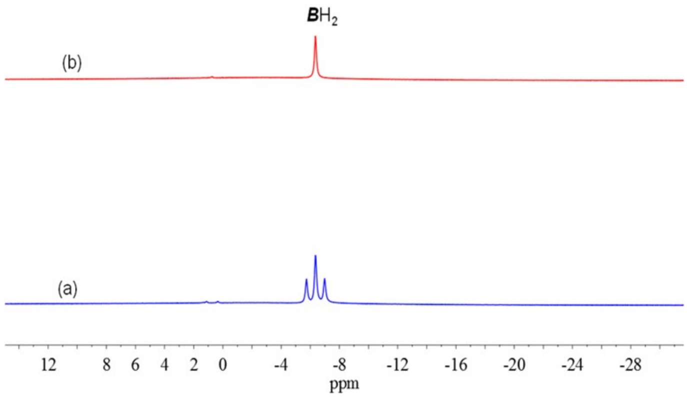

Fig. S6. ^{11}B (a) and $^{11}B\{^1H\}$ NMR (b) spectra of $CH_3NH_2BH_2Cl$ (1b) in THF.

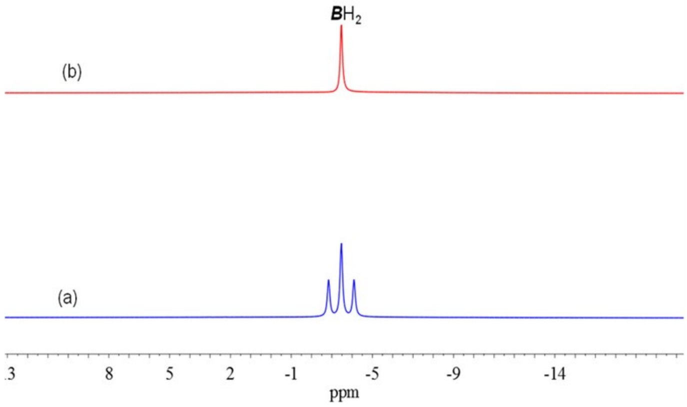

Fig. S7. ^{11}B (a) and $^{11}B\{^1H\}$ NMR (b) spectra of $(CH_3)_2NHBH_2Cl$ (1c) in THF.

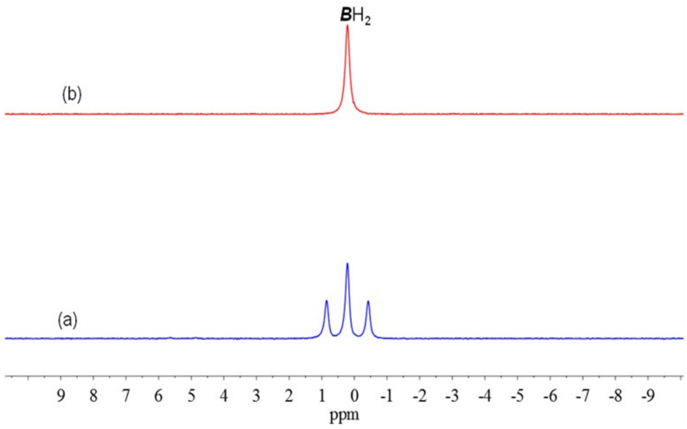

Fig. S8. ^{11}B (a) and $^{11}B\{^1H\}$ NMR (b) spectra of $(CH_3)_3NBH_2Cl$ (1d) in THF.

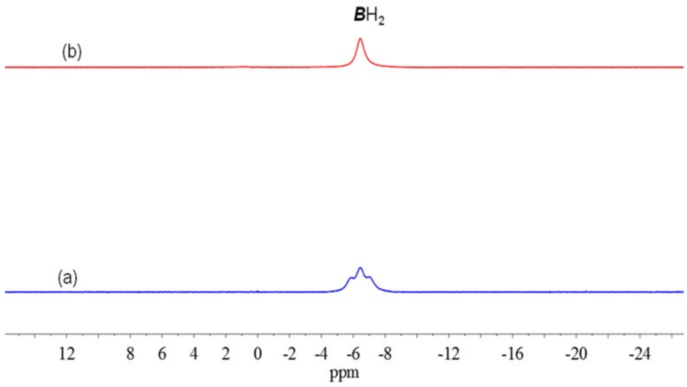

Fig. S9. ^{11}B (a) and $^{11}B\{^1H\}$ NMR (b) spectra of $ClBH_2NH_2CH_2CH_2NH_2BH_2Cl$ (1e) in THF.

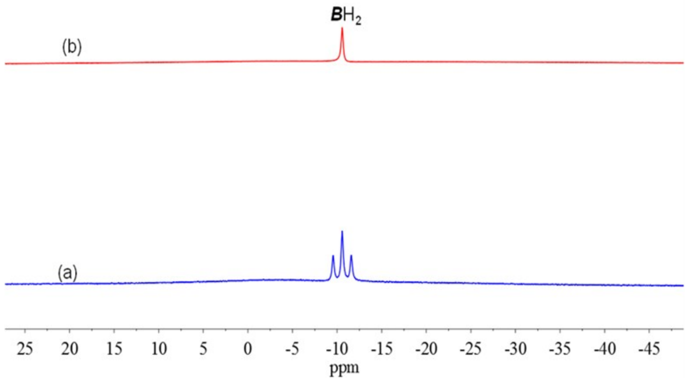

Fig. S10. ^{11}B (a) and $^{11}B\{^1H\}$ NMR (b) spectra of $(CH_3)_3NBH_2I$ (1f) in toluene.

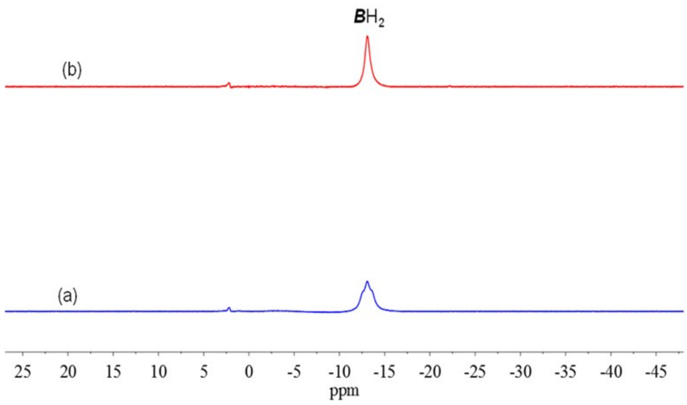

Fig. S11. ^{11}**B (a) and** ^{11}**B{**^{1}**H} NMR (b) spectra of** $(NH_3)_2BH_2Cl$ **(1g) in DMF.**

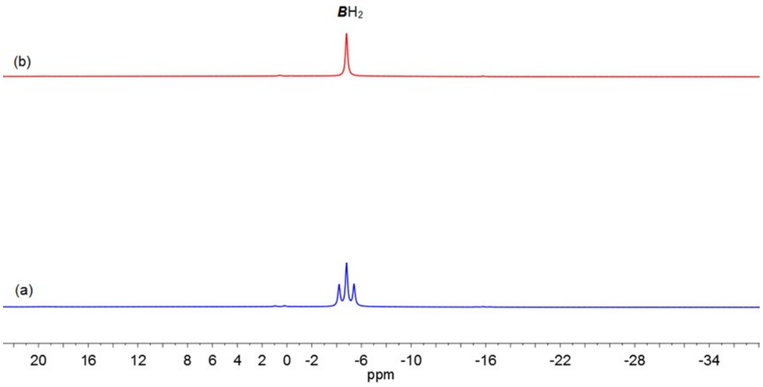

Fig. S12. ^{11}**B (a) and** ^{11}**B{**^{1}**H} NMR (b) spectra of** $NH_3BH_2C(NO_2)_3$ **(3a) in** CD_3CN**.**

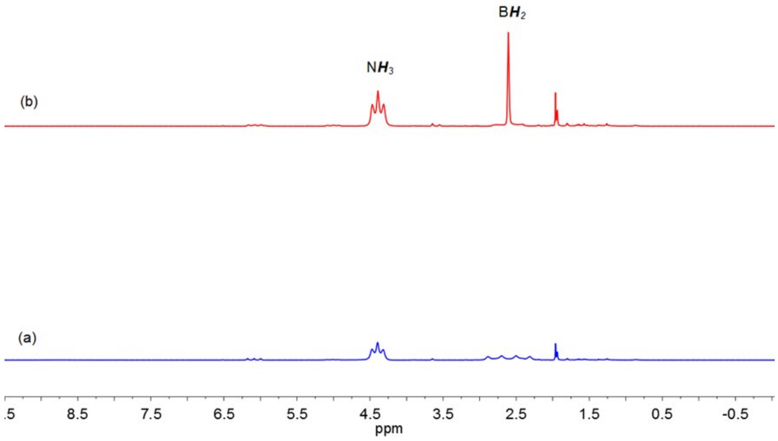

Fig. S13. ^{1}**H (a) and** ^{1}**H{**^{11}**B} NMR (b) spectra of** $NH_3BH_2C(NO_2)_3$ **(3a) in** CD_3CN**.**

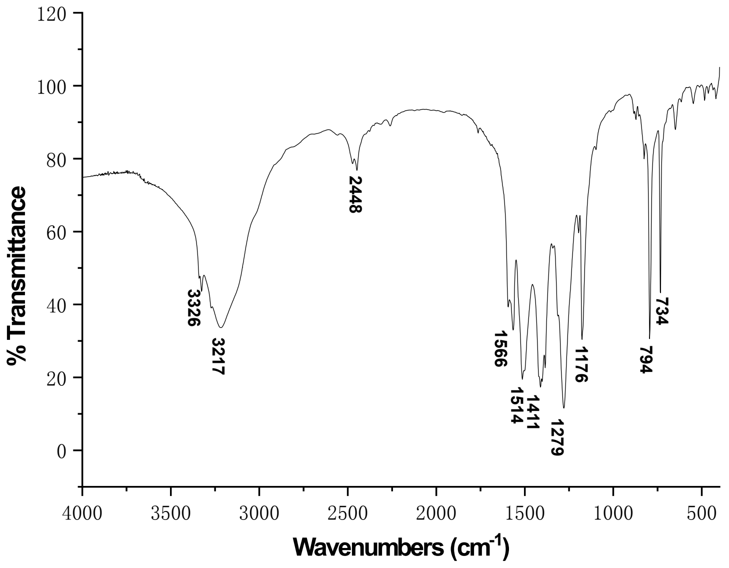

Fig. S14. IR spectrum of $NH_3BH_2C(NO_2)_3$ (3a).

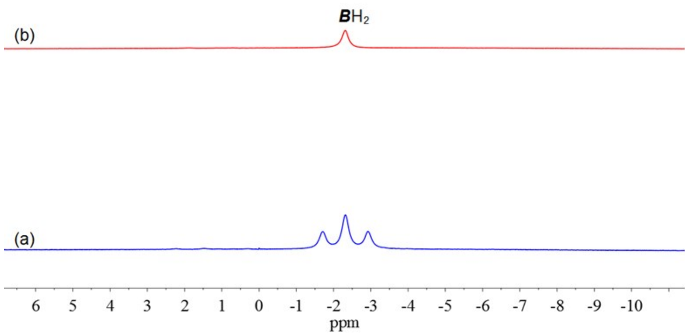

Fig. S15. ^{11}B (a) and $^{11}B\{^1H\}$ NMR (b) spectra of $CH_3NH_2BH_2C(NO_2)_3$ (3b) in CD_3CN.

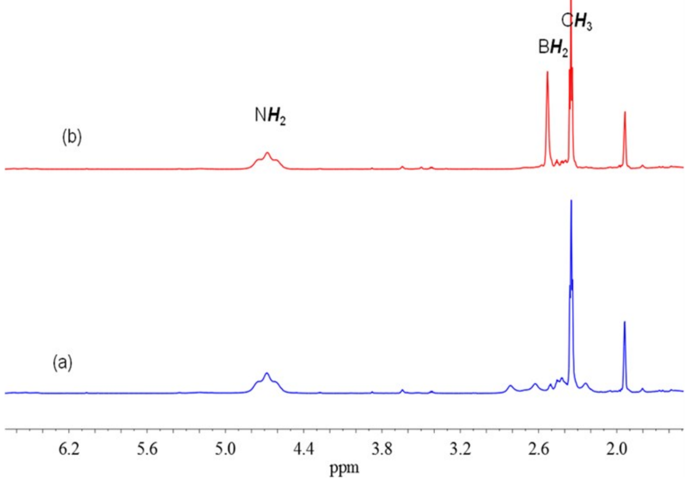

Fig. S16. ^1H (a) and $^1H\{^{11}B\}$ NMR (b) spectra of $CH_3NH_2BH_2C(NO_2)_3$ (3b) in CD_3CN.

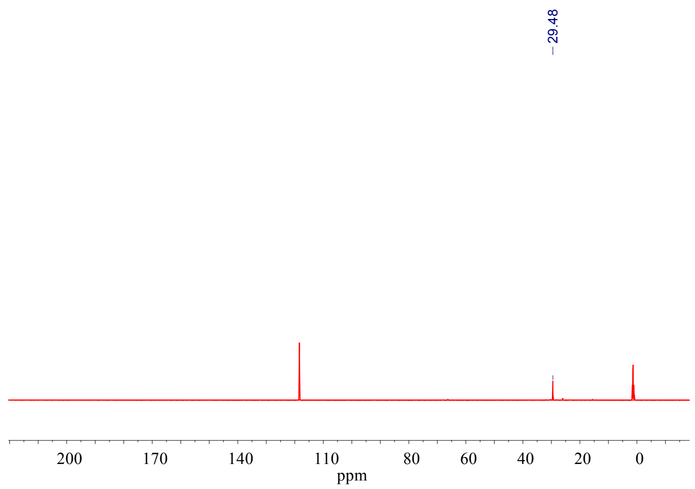

Fig. S17. ^{13}C NMR spectrum of $CH_3NH_2BH_2C(NO_2)_3$ (3b) in CD_3CN.

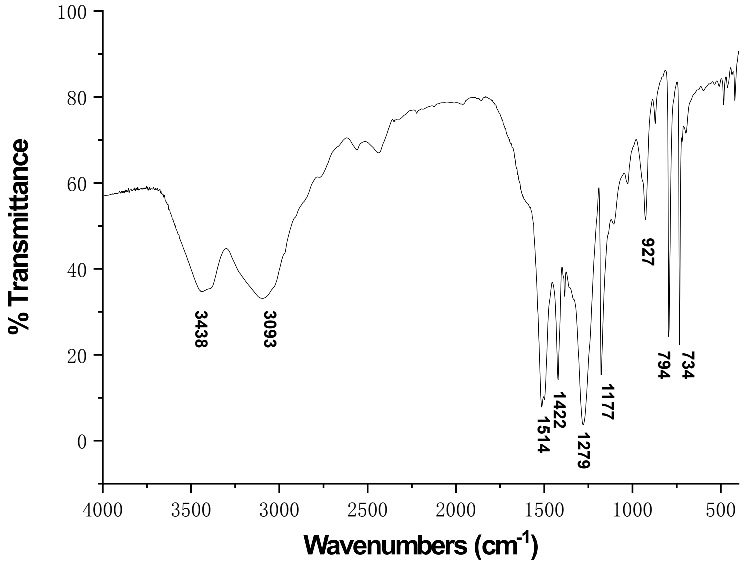

Fig. S18. IR spectrum of $CH_3NH_2BH_2C(NO_2)_3$ (3b).

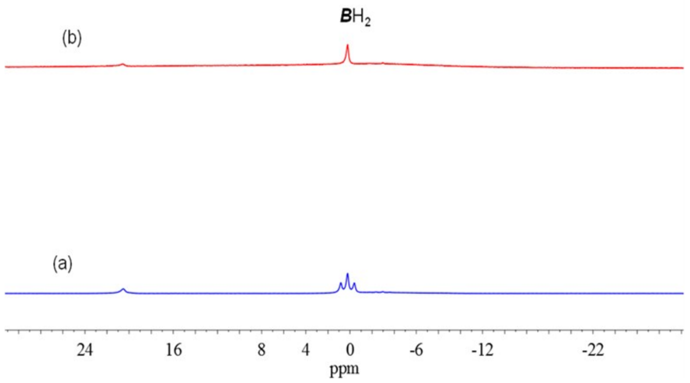

Fig. S19. ^{11}B (a) and $^{11}B\{^1H\}$ NMR (b) spectra of $(CH_3)_2NHBH_2C(NO_2)_3$ (3c) in CD_3CN.

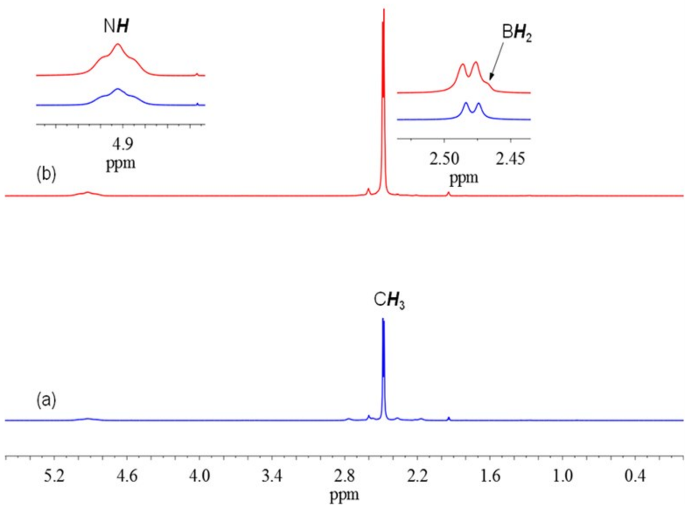

Fig. S20. ^{1}H **(a) and** $^{1}H\{^{11}B\}$ **NMR (b) spectra of** $(CH_3)_2NHBH_2C(NO_2)_3$ **(3c) in** CD_3CN**.**

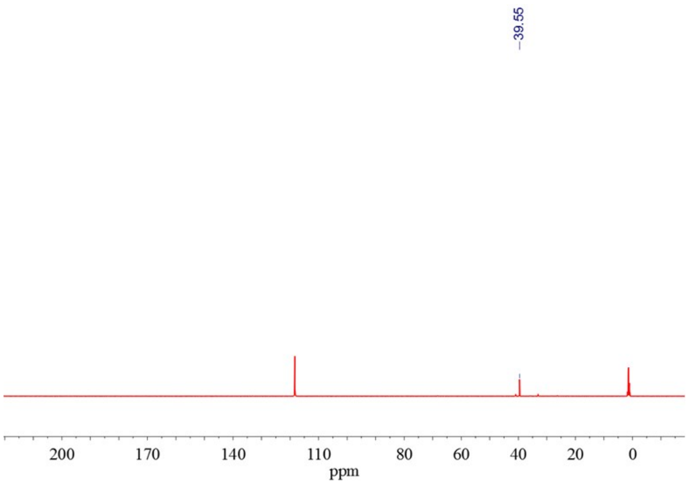

Fig. S21. ^{13}C **NMR spectrum of** $(CH_3)_2NHBH_2C(NO_2)_3$ **(3c) in** CD_3CN**.**

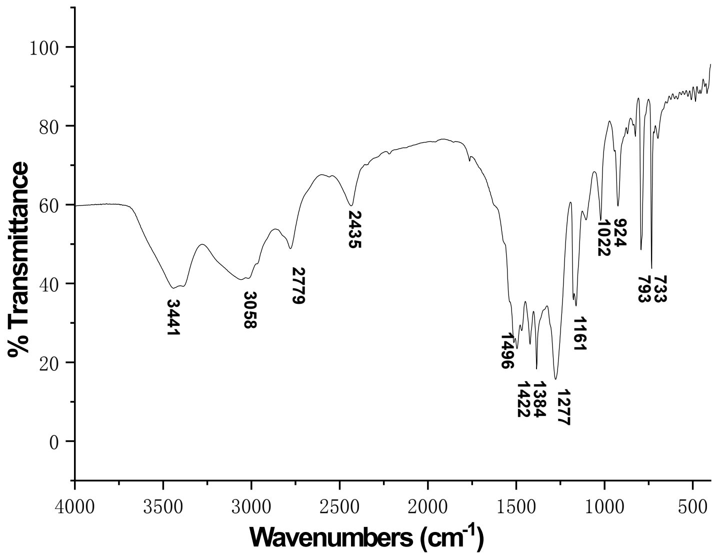

Fig. S22. IR spectrum of $(CH_3)_2NHBH_2C(NO_2)_3$ (3c).

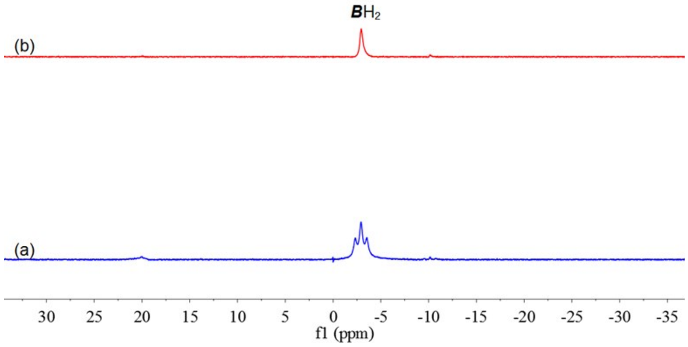

Fig. S23. ^{11}B (a) and $^{11}B\{^1H\}$ NMR (b) spectra of $[CH_2NH_2BH_2C(NO_2)_3]_2$ (3e) in CD_3CN.

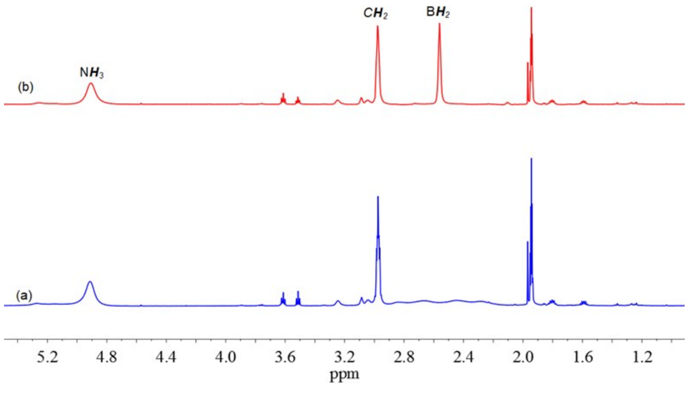

Fig. S24. ^1H (a) and $^1H\{^{11}B\}$ NMR (b) spectra of $[CH_2NH_2BH_2C(NO_2)_3]_2$ (3e) in CD_3CN.

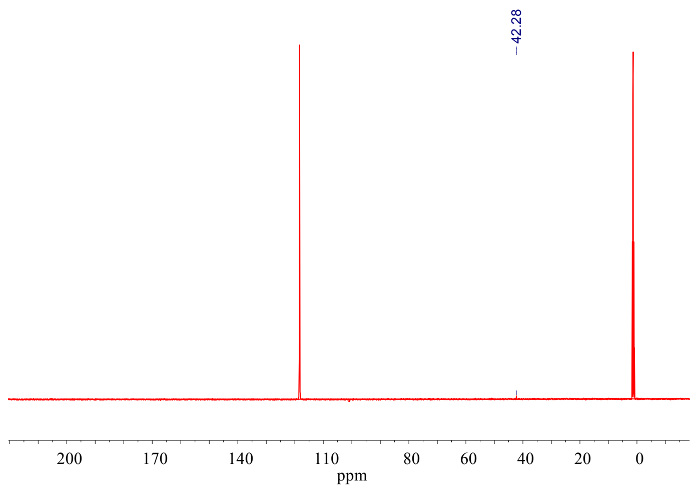

Fig. S25. ^{13}**C NMR spectrum of** $[CH_2NH_2BH_2C(NO_2)_3]_2$ **(3e) in** CD_3CN**.**

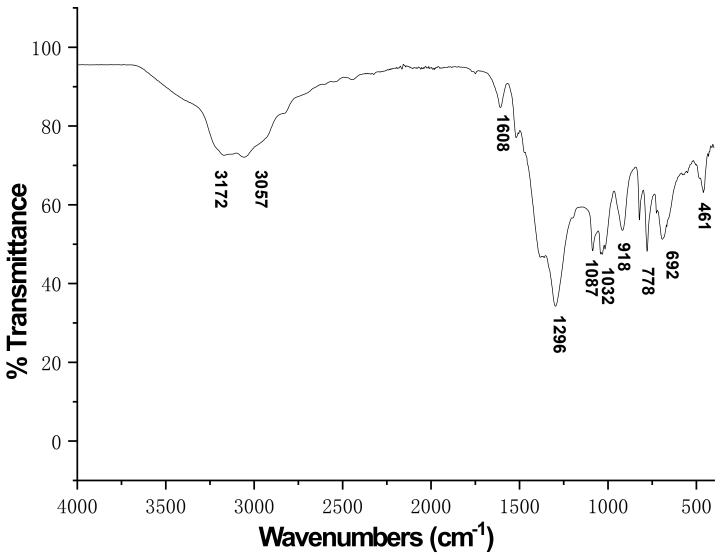

Fig. S26. IR spectrum of $[CH_2NH_2BH_2C(NO_2)_3]_2$ **(3e).**

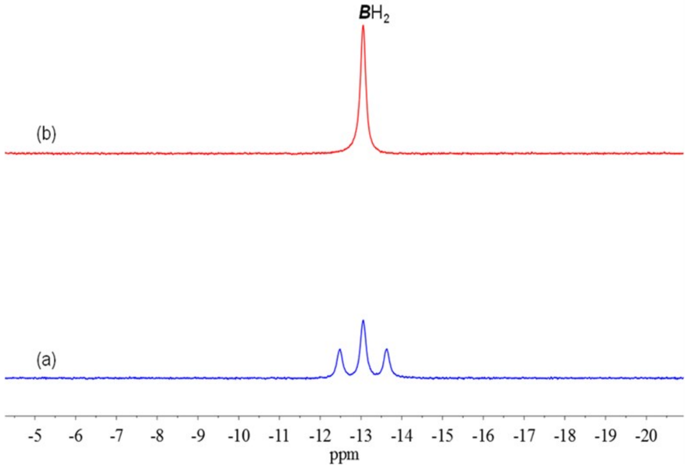

Fig. S27. ^{11}B (a) and ^{11}B{^{1}H} NMR (b) spectra of $[H_2B(NH_3)_2][C(NO_2)_3]$ (3g) in CD_3CN.

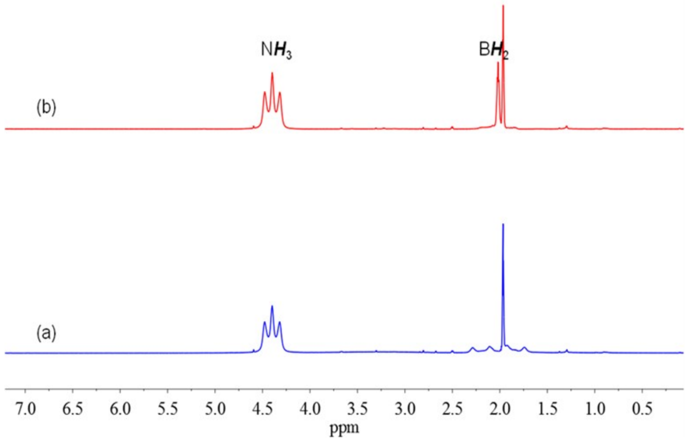

Fig. S28. ^{1}H (a) and ^{1}H{^{11}B} NMR (b) spectra of $[H_2B(NH_3)_2][C(NO_2)_3]$ (3g) in CD_3CN.

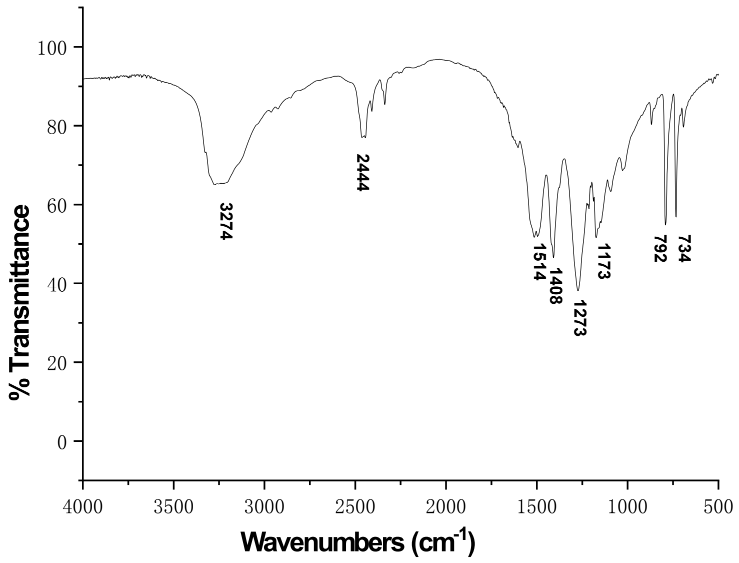

Fig. S29. IR spectrum of $[H_2B(NH_3)_2][C(NO_2)_3]$ (3g).

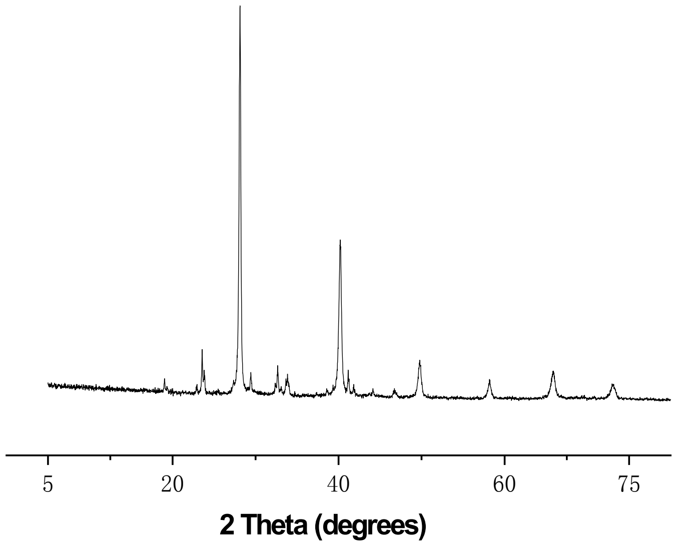

Fig. S30. X-ray powder diffraction pattern of KCl.

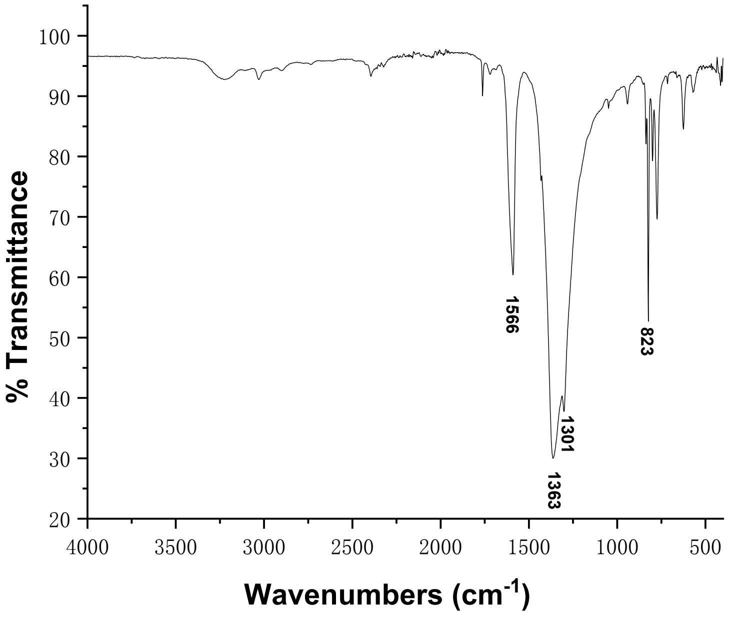

Fig. S31. IR spectrum of $K[C(NO_2)_3]$.
